# ICTV Virus Taxonomy Profile: *Amnoonviridae* 2023

**DOI:** 10.1099/jgv.0.001903

**Published:** 2023-10-24

**Authors:** Eugene V. Koonin, Mart Krupovic, Win Surachetpong, Yuri I. Wolf, Jens H. Kuhn

**Affiliations:** ^1^​ National Center for Biotechnology Information, National Library of Medicine, National Institutes of Health, Bethesda MD 20894, USA; ^2^​ Institut Pasteur, Université Paris Cité, Archaeal Virology Unit, Paris 75015, France; ^3^​ Department of Veterinary Microbiology and Immunology, Faculty of Veterinary Medicine, Kasetsart University, Bangkok, Thailand; ^4^​ Integrated Research Facility at Fort Detrick, National Institute of Allergy and Infectious Diseases, National Institutes of Health, Fort Detrick, Frederick, MD 21702, USA

**Keywords:** ICTV Report, taxonomy, *Amnoonviridae*, tilapinevirus, tilapia lake virus

## Abstract

*Amnoonviridae* is a family of negative-sense RNA viruses with genomes totalling about 10.3 kb. These viruses have been found in fish. The amnoonvirid genome consists of 10 segments, each with at least 1 open reading frame (ORF). The RNA1–3 ORFs encode the three subunits of the viral polymerase. The RNA4 ORF encodes a nucleoprotein. This is a summary of the International Committee on Taxonomy of Viruses (ICTV) Report on the family *Amnoonviridae*, which is available at ictv.global/report/amnoonviridae.

## Virion

Amnoonvirids produce sphercial, slightly pleomorphic (likely enveloped) particles 55–100 nm in diameter [1, 2] (Table 1).

**Table 1.** Characteristics of members of the family *Amnoonviridae*

Example	tilapia lake virus (KU751814–KU751823), species *Tilapinevirus tilapiae*, genus *Tilapinevirus*
Virion	Enveloped, spherical; 55–100 nm
Genome	About 10.3 kb of decasegmented negative-sense RNA
Replication	Unknown
Translation	Unknown
Host range	Actinopterygiid fish
Taxonomy	Realm *Riboviria*, kingdom *Orthornavirae*, phylum *Negarnaviricota*, class *Insthoviricetes*, order *Articulavirales*; the family includes the genus *Tilapinevirus* and the species *Tilapinevirus tilapiae*

## Genome

**Fig. 1.**
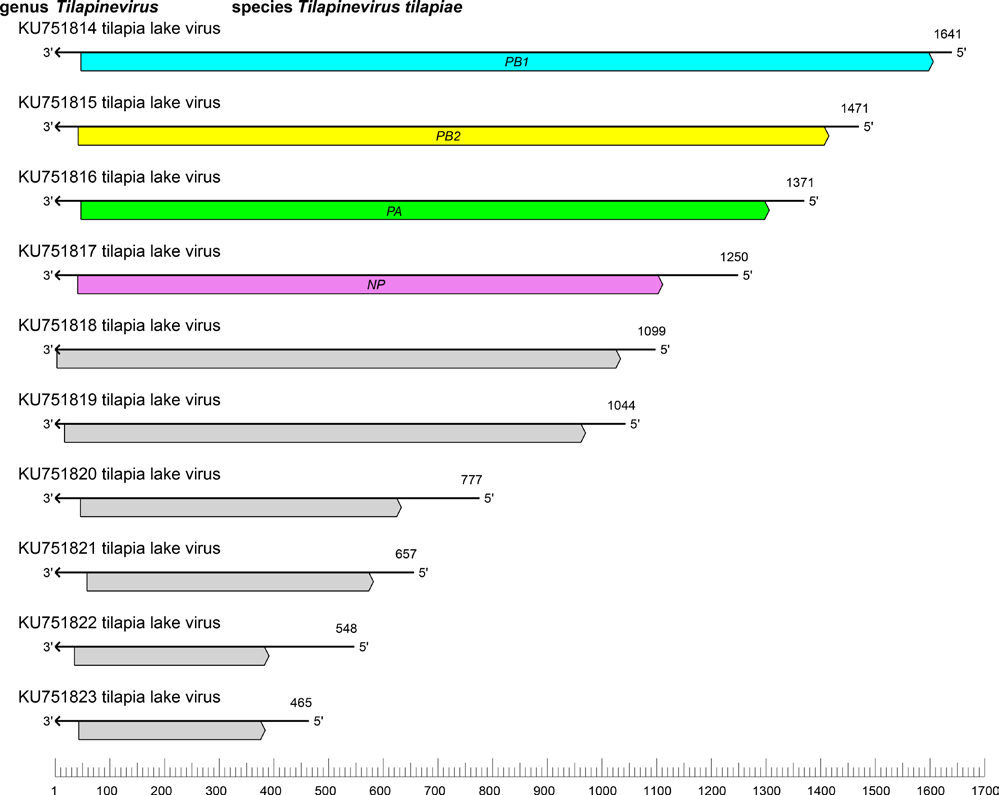
Genome organisation of tilapia lake virus. ORFs are coloured according to the predicted protein function (NP, nucleoprotein gene; PA, polymerase subunit 3 gene; PB1, polymerase subunit 1 gene encoding an RdRP domain; PB2, polymerase subunit 2 gene). Genes of unknown function are coloured grey.

The amnoonvirid genome comprises 10 segments of linear negative-sense RNA with a total length of of about 10.3 kb (tilapia lake virus – RNA1 :1641 nt; RNA2 : 1471 nt; RNA3 :1371 nt; RNA4 : 1250 nt; RNA5 :1099 nt; RNA6 : 1044 nt; RNA7 : 777 nt; RNA8 :657 nt; RNA9 : 548 nt; and RNA10 : 465 nt) (Fig. 1). All 10 segments have conserved, complementary sequences at their 5′- and 3′-ends. The RNA1 ORF encodes a protein with an RNA-directed RNA polymerase (RdRP) domain homologous to the PB1 of influenza C virus (*Orthomyxoviridae*: *Gammainfluenzavirus*) [3–5]. The RNA2 and RNA3 ORFs encode the PB2 and PA polymerase subunit homologues of orthomyxovirids [6]. The RNA4 ORF encodes a nucleoprotein [7].

## Replication

Cell entry occurs via dynamin-mediated endocytosis in a cholesterol-dependent, cytoskeleton-dependent manner that is independent of clathrin and pH [8].

## Pathogenicity

Tilapia lake virus is highly virulent in tilapia, with systemic infection involving most organs. Infection results in lethargy, ocular disease (such as cataracts, endophthalmitis or exophthalmos), skin erosions and haemorrhages, and severe anaemia, leading to death in >80 % of cases [1].

## Taxonomy

**Fig. 2.**
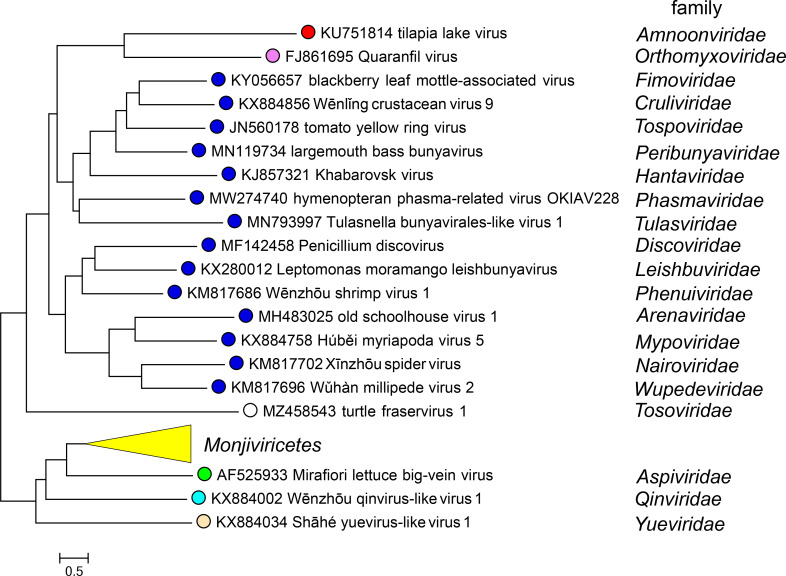
Phylogenetic relationships of the RdRP core domain of viruses in the phylum *Negarnaviricota*. For full details see *Amnoonviridae* ICTV Report.

Current taxonomy: ictv.global/taxonomy. The family *Amnoonviridae* includes the genus *Tilapinevirus* for one species of viruses that infect fish. Viruses in the family *Amnoonviridae* are most closely related to articulaviral orthomyxovirids [4] (Fig. 2).

## Resources

Full ICTV Report on the family *Amnoonviridae*: ictv.global/report/amnoonviridae.
